# Structured Differential Diagnosis of Orofacial Pain Associated with an Enamel Crack Using ICOP and ICHD-3: A Case Report

**DOI:** 10.3390/healthcare14132001

**Published:** 2026-07-06

**Authors:** Kohei Shimizu, Takuya Yasukawa, Masayuki Okano, Aki Kawamoto, Noboru Noma, Makoto Hayashi, Osamu Takeichi

**Affiliations:** 1 Department of Endodontics, Nihon University School of Dentistry, Tokyo 101-8310, Japan; yasukawa.takuya@nihon-u.ac.jp (T.Y.); dema23006@g.nihon-u.ac.jp (M.O.); hayashi.makoto53@nihon-u.ac.jp (M.H.); takeichi.osamu@nihon-u.ac.jp (O.T.); 2 Division of Advanced Dental Treatment, Dental Research Center, Nihon University School of Dentistry, Tokyo 101-8310, Japan; 3 Dental Hygienist Section, Nihon University School of Dentistry Dental Hospital, Tokyo 101-8310, Japan; kawamoto.aki@nihon-u.ac.jp; 4 Department of Oral Medicine, Nihon University School of Dentistry, Tokyo 101-8310, Japan; noma.noboru@nihon-u.ac.jp; 5 Division of Clinical Research, Dental Research Center, Nihon University School of Dentistry, Tokyo 101-8310, Japan

**Keywords:** cracked tooth syndrome, endodontic diagnosis, orofacial pain, referred pain, diagnostic local anaesthesia, International Classification of Orofacial Pain (ICOP), International Classification of Headache Disorders (ICHD-3), vital pulp therapy, differential diagnosis, cone-beam computed tomography

## Abstract

**Highlights:**

**What are the main findings?**

- An enamel crack in a mandibular first molar presented with atypical symptoms, including referred temporal pain that mimicked non-odontogenic pain conditions.
- A structured diagnostic approach integrating ICOP, ICHD-3, CBCT, pulp sensibility testing, and diagnostic local anaesthesia supported identification of the most likely odontogenic pain source.

**What are the implications of the main findings?**

- Cracked teeth should be considered in the differential diagnosis of atypical tooth-related pain accompanied by headache-like or referred pain symptoms.
- Conservative crack sealing may allow symptom resolution and preservation of pulp vitality in carefully selected cases without immediate endodontic treatment.

**Abstract:**

**Background:** Cracked teeth may present with variable and atypical symptoms, sometimes mimicking non-odontogenic orofacial pain conditions, making diagnosis challenging, particularly when cracks appear limited to enamel. **Case presentation:** A 36-year-old woman presented with intermittent pain in a mandibular molar radiating to the ipsilateral temporal region. Clinical examination identified a crack line on the lingual surface of the mandibular first molar. Pulp sensibility testing (cold test and electric pulp test), occlusal loading tests, and cone-beam computed tomography (CBCT) were performed. CBCT was used primarily to exclude vertical root fracture and periapical pathology, and no radiographic abnormalities were identified. Differential diagnosis was conducted using structured diagnostic frameworks, including the International Classification of Orofacial Pain and the International Classification of Headache Disorders (3rd edition). Diagnostic local anaesthesia eliminated both biting pain and referred pain, supporting an odontogenic source. Collectively, the findings suggested that the enamel crack was the most likely source of odontogenic pain, although a definitive causal relationship could not be established. Because pulp sensibility remained normal, conservative management was selected. Crack sealing with a methyl methacrylate-based adhesive resin resulted in complete symptom resolution that was maintained throughout a 3-year follow-up period without the need for root canal treatment. **Conclusions:** Although the diagnosis remained probabilistic, the structured diagnostic approach, together with the favourable clinical response after crack sealing, supported the enamel crack as the most likely source of odontogenic pain.

## 1. Introduction

Toothache in the oral and maxillofacial region can broadly be categorized as odontogenic or non-odontogenic. In general dental practice, most cases are odontogenic, whereas mixed odontogenic and non-odontogenic pain accounts for less than 10% of cases worldwide [1]. Among odontogenic conditions, cracked crowns and fractured teeth are particularly prone to misdiagnosis because symptoms are often unstable, fluctuating and atypical, thereby mimicking non-odontogenic disorders such as temporomandibular disorders, sinusitis or other orofacial pain conditions [2]. When a crack line is confined to enamel or superficial dentine, establishing a causal relationship between the structural defect and symptoms remains challenging [3,4,5]. Evidence regarding symptomatic enamel cracks without dentinal involvement remains limited, and their clinical significance has not been fully established [3,4,5].

Diagnostic modalities such as thermal testing, occlusal loading tests, microscopy and cone-beam computed tomography (CBCT) may provide supportive information but are not always definitive [3,4,5]. The International Classification of Orofacial Pain (ICOP) provides a structured framework for diagnosing orofacial pain [6,7]. Similarly, the International Classification of Headache Disorders (ICHD-3) provides standardized criteria that may assist in the exclusion of headache-related conditions presenting with referred facial pain [8].

**Figure 1:**
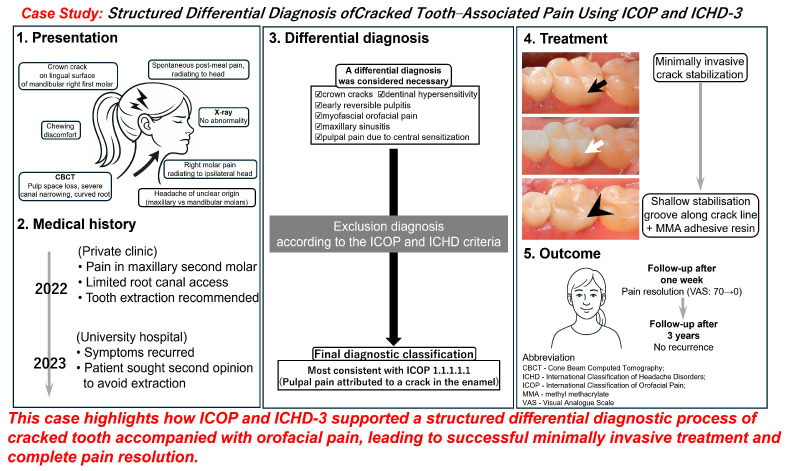
Schematic diagram summarizing the present case report. **Abbreviations:** CBCT—cone-beam computed tomography; ICHD—International Classification of Headache Disorders; ICOP—International Classification of Orofacial Pain; MMA—methyl methacrylate; VAS—visual analogue scale.

This report describes a diagnostically challenging case of tooth pain associated with an enamel crack accompanied by referred temporal pain. The combined application of ICOP, ICHD-3 and diagnostic local anaesthetic testing facilitated a structured differential diagnostic process and guided conservative management. A schematic diagram summarizing the present case report is shown in Figure 1.

## 2. Case Presentation

### 2.1. Ethical Considerations

This case report was prepared in accordance with the CARE guidelines of the EQUATOR Network [9]. Ethical approval was obtained from the institutional ethics committee (approval numbers: EP22D016, approved 19 January 2023; EP24D018, approved 16 January 2025). All procedures were conducted in accordance with the Declaration of Helsinki. Written informed consent was obtained from the patient for publication of this case report and accompanying clinical images.

### 2.2. Patient Information

A 36-year-old Ukrainian woman residing in Japan presented to her referring dentist with pain in the right molar region radiating to the ipsilateral side of the head. She reported psychological stress related to the conflict in her home country. At the referring clinic, radiographs showed no clear pathology. Antibiotics and nonsteroidal anti-inflammatory drugs were ineffective. Because the pain was poorly localized and radiated to the head, both odontogenic and non-odontogenic causes were considered. The second molar was suspected, but severe canal calcification made treatment difficult and extraction was recommended. The patient sought further evaluation. Medical history was unremarkable. Psychosocial screening revealed moderate anxiety (GAD-7: 11) and mild depression (PHQ-9: 7) [10,11]. The Oral Behaviour Checklist identified parafunctional habits including clenching [12,13]. These findings were considered during the differential diagnostic process because they may influence pain perception and symptom presentation.

### 2.3. Clinical Findings

**Figure 2:**
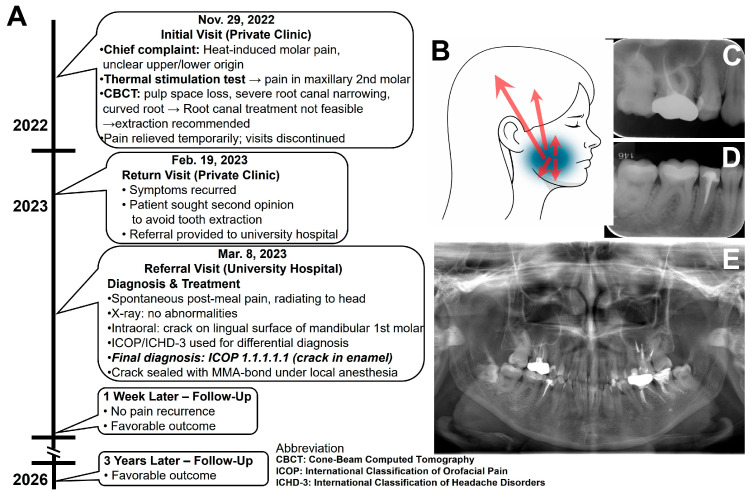
(**A**) Timeline of interventions and corresponding outcomes; (**B**) The patient reported spontaneous non-persistent pain symptoms occurring occasionally after eating, originating from the maxillary and mandibular molar tooth regions and referring to the ipsilateral side of the head; (**C**–**E**) Dental and panoramic radiographs showing no significant findings.

**Figure 3:**
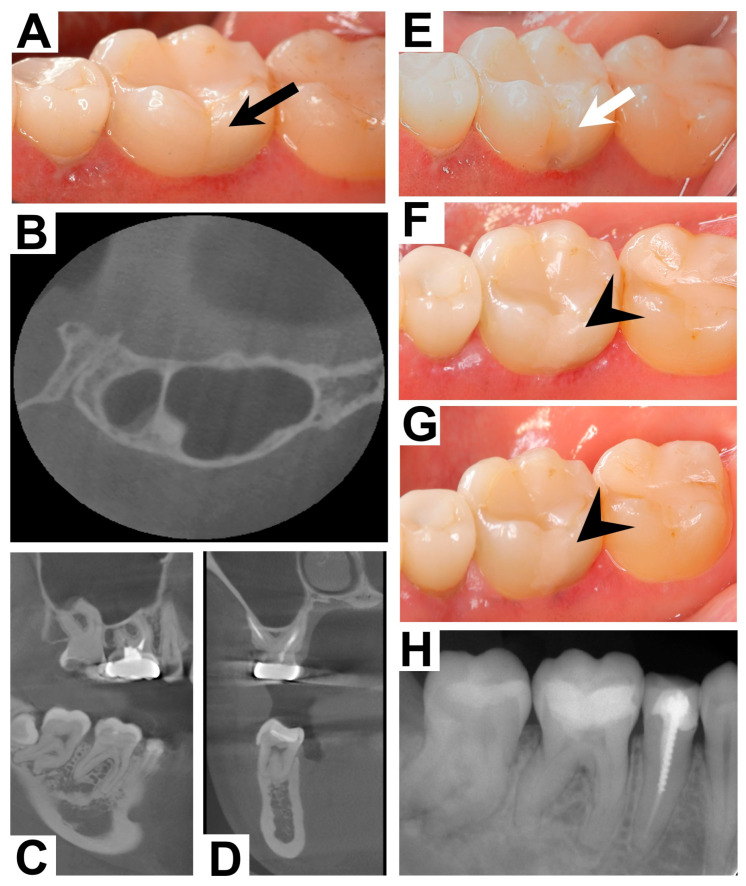
(**A**) An intraoral photograph of the crack line in the lingual surface of the right mandibular first molar (black arrow); (**B**–**D**) The cone-beam computed tomography (CBCT) images of the right mandibular first molar and right maxillary sinus; (**E**) Following the administration of local anaesthesia, a diamond bur was used to prepare a shallow groove along the crack line to facilitate adhesion (white arrow). (**F**) After appropriate acid etching, a methyl methacrylate (MMA)-based dental adhesive material (Super-bond; Sun Medical Co., Ltd., Shiga, Japan) was used to fill the groove (arrow head); (**G**,**H**) Patient’s prognosis monitored regularly (arrow head), demonstrating favourable progression and absence of pain recurrence (3 years later).

Figure 2A shows the timeline of interventions and corresponding outcomes. The patient reported intermittent spontaneous pain after meals radiating to the temporal region (Figure 2B). Periapical and panoramic radiographs showed no periapical pathology (Figure 2C–E). Intraoral examination revealed a crack line on the lingual crown surface of the mandibular first molar (Figure 3A). CBCT was performed to evaluate possible structural pathology and demonstrated no evidence of vertical root fracture, periapical disease, or pulpal involvement (Figure 3B,C,E). No swelling or sinus tract formation was observed clinically. Periodontal examination revealed probing depths of ≤3 mm, no bleeding on probing, no pathological tooth mobility, no periodontal pockets, and no furcation involvement. Occlusal examination revealed no premature contacts or traumatic occlusal interference.

Percussion and occlusal loading were negative, although thermal stimulation produced mild discomfort. Cold testing was performed using PULPER^®^ (GC Corporation, Tokyo, Japan), and pulp sensibility was assessed using an electric pulp tester (Vitality Scanner; SybronEndo, Orange, CA, USA). Both tests demonstrated normal pulp sensibility. Differential diagnoses included cracked tooth, dentine hypersensitivity, reversible pulpitis, myofascial pain, sinus-related pain, and headache-related disorders. To improve diagnostic transparency and reproducibility, a structured diagnostic pathway integrating clinical findings, pulp sensibility testing, CBCT, diagnostic local anaesthesia, ICOP, and ICHD-3 criteria was applied (Table 1).

Primary headache disorders and sinus-related pain were excluded based on ICHD-3 criteria, clinical findings, imaging findings, and the response to diagnostic local anaesthesia. The diagnostic assessment was guided by ICOP and ICHD-3 criteria. Diagnostic local anaesthetic infiltration was administered in the buccal vestibule adjacent to the mandibular first molar using EPILIDO^®^ (2% lidocaine with 1:80,000 epinephrine; Nipro Corporation, Osaka, Japan). The procedure temporarily eliminated occlusal pain, thermal pain, and the referred temporal pain, supporting an odontogenic source. Based on the collective clinical findings and structured diagnostic assessment, the enamel crack was considered the most likely contributor to the patient’s symptoms (ICOP 1.1.1.1.1).

### 2.4. Treatment

Because pulp vitality was preserved and no clinical or radiographic evidence of pulpal or periapical disease was identified, conservative management was selected. A shallow groove was prepared along the crack line to facilitate sealing (Figure 3E). After phosphoric acid etching with Enamel Etchant Gel (Sun Medical, Moriyama, Japan), the groove was sealed using a methyl methacrylate-based adhesive resin (Super-Bond^®^, Sun Medical, Moriyama, Japan) according to the manufacturer’s instructions. The groove was then restored using a tooth-colored methyl methacrylate-based adhesive resin (Super-Bond, Sun Medical, Moriyama, Japan) (Figure 3F). No additional composite resin restoration was placed because the primary objective of treatment was crack sealing rather than reinforcement of tooth structure, and the tooth-colored Super-Bond was considered sufficient for this purpose. The patient was informed that further treatment options, including indirect restoration, endodontic treatment, or extraction, would be considered if symptoms persisted or worsened.

### 2.5. Outcome

Follow-up assessments were performed at 3–6-month intervals over a 3-year period. The patient’s visual analogue scale (VAS) score for tooth-related pain decreased from 70 to 0 within one week after treatment and remained unchanged throughout follow-up. Tooth-related pain and associated sleep disturbance resolved completely. Pulp sensibility remained normal, and no intraoral or radiographic abnormalities were observed during follow-up (Figure 3G,H).

The favorable clinical outcome was maintained throughout the 3-year observation period without the need for endodontic treatment or extraction.

## 3. Discussion

Cracked teeth are frequently misdiagnosed because symptoms may be intermittent and mimic non-odontogenic pain [3,14]. When cracks are limited to enamel or superficial dentine, determining causation is particularly difficult [3,5]. Diagnostic tools such as thermal testing, occlusal loading and CBCT may assist but may not establish definitive diagnosis [6,15]. Although classic cracked tooth syndrome is commonly associated with pain during biting or upon release of biting pressure, occlusal loading was negative in the present case. This discrepancy may be explained by the superficial location of the enamel crack, intermittent symptom expression, or the direction of functional loading relative to the crack orientation. The structured diagnostic pathway described in Table 1 improved transparency and reproducibility of the diagnostic process and facilitated differential diagnosis using both ICOP and ICHD-3 criteria. Management strategies vary [15]. Conservative sealing may produce favourable outcomes in selected cases [3,15], whereas deeper cracks involving dentine or pulp are more likely to require endodontic treatment [3,16]. In the present case, methyl methacrylate-based adhesive resin was selected because the crack appeared to be limited to enamel, pulp sensibility was preserved, and there was no clinical or radiographic evidence of pulpal or periapical disease. Additional composite restoration or cuspal coverage was not performed because the primary treatment objective was sealing of a superficial crack rather than structural reinforcement. This conservative approach is consistent with previous reports suggesting that adhesive sealing may be effective for carefully selected cracked teeth without pulpal involvement [3,15,16]. However, this approach should be limited to carefully selected cases and requires long-term follow-up, with escalation to indirect restoration, endodontic treatment, or extraction if symptoms persist or structural progression is suspected.

The precise mechanism underlying symptom generation in the present case cannot be determined with certainty. Several factors may have contributed to symptom development. Previous studies have suggested that cracks and structural defects in teeth may influence stress distribution and sensory responses within the dentine–pulp complex [17,18,19]. However, whether such mechanisms apply to enamel cracks alone remains uncertain. In addition, psychological stress and parafunctional habits may have increased occlusal loading and influenced symptom presentation. Anxiety, depression, and parafunctional habits may have amplified pain perception or contributed to symptom persistence. However, the immediate elimination of symptoms following diagnostic local anaesthesia and sustained symptom resolution after crack sealing suggest that these factors were secondary rather than primary contributors. Therefore, they were considered modulatory factors rather than the principal source of pain in this case. Elimination of both local and referred pain following diagnostic local anaesthesia supported an odontogenic contribution to the symptoms. Furthermore, the favorable clinical response following crack sealing suggests that the enamel crack was a plausible contributing factor. Nevertheless, this interpretation should be made with caution because diagnostic local anaesthesia and symptom resolution after crack sealing support—but do not definitively prove—a causal relationship. Alternative explanations, including spontaneous symptom fluctuation or modulation by psychosocial and parafunctional factors, cannot be completely excluded in a single case report. This case highlights the importance of comprehensive differential diagnosis and careful case selection when considering conservative management of cracked teeth.

## 4. Limitations

This report has several limitations. First, CBCT has limited ability to detect enamel and dentin cracks and therefore could not be used to definitively confirm the extent of the crack. Second, a causal relationship between the enamel crack and the patient’s symptoms cannot be conclusively established from a single case report. Third, psychological stress and parafunctional habits may have influenced symptom perception and clinical presentation. Detailed occlusal analysis was not quantitatively performed; therefore, subtle occlusal interferences cannot be completely excluded. Finally, ICOP lacks a specific category for sinus-related toothache, requiring supplementary consideration of ICHD-3 criteria during the diagnostic process.

## 5. Conclusions

**Figure 4:**
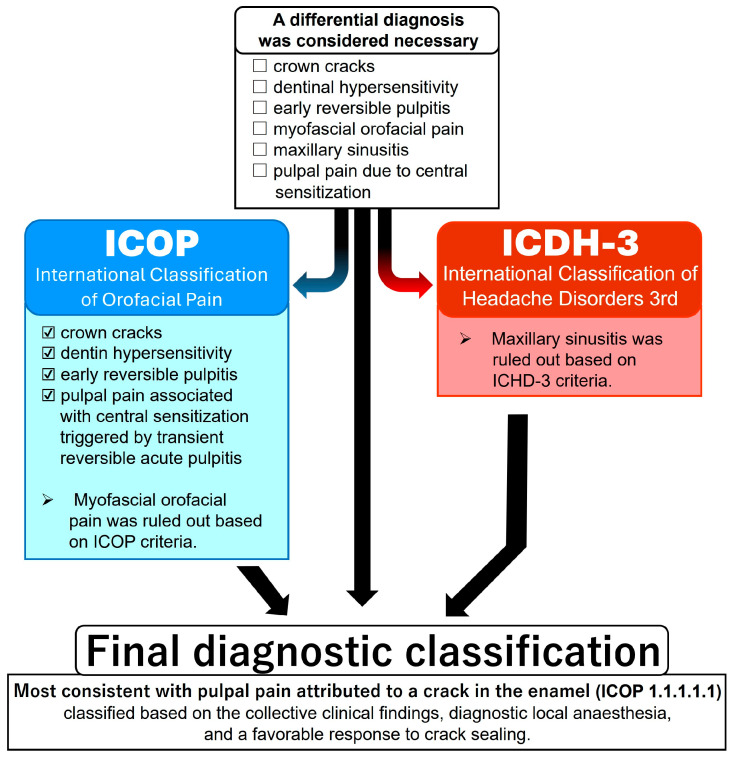
Diagnostic flowchart illustrating the structured differential diagnostic process integrating ICOP and ICHD-3, supporting identification of the most likely odontogenic pain source and classification as ICOP 1.1.1.1.1.

This case demonstrates the usefulness of integrating ICOP and ICHD-3 within a structured differential diagnostic approach for patients presenting with atypical tooth-related pain. Diagnostic local anaesthesia and comprehensive clinical assessment supported conservative management, resulting in preservation of pulp vitality and long-term symptom resolution. Although these findings supported the enamel crack as the most likely pain source, a definitive causal relationship cannot be established from a single case report. Clinicians should consider cracked teeth as a potential source of referred orofacial pain and carefully evaluate such cases before undertaking irreversible treatment (Figure 4).

## 6. Patient Perspective

After treatment with Super-Bond, the tooth pain I had previously experienced disappeared. Due to ongoing stress related to the war in my home country, I noticed clenching during sleep and awake-time tooth-contacting habits and occasionally experienced sensitivity to cold water, which improved with the use of desensitizing toothpaste. Although headaches still occurred occasionally, their frequency decreased markedly after a night guard was provided, and they have almost completely resolved since October 2023.

## Figures and Tables

**Table 1:** Structured diagnostic reasoning process integrating ICOP and ICHD-3.

Diagnostic Consideration	Supporting Findings	Reason for Exclusion/Inclusion	Diagnostic Framework
Primary headache disorder	Temporal pain present	ICHD-3 criteria not fulfilled; symptoms abolished by local anaesthesia	ICHD-3
Sinus-related headache/facial pain	Referred temporal pain	No sinonasal symptoms, no radiographic evidence; symptoms abolished by local anaesthesia	ICHD-3
Myofascial pain	Referred pain pattern	Clinical findings inconsistent; local anaesthesia eliminated symptoms	ICOP differential diagnosis
Reversible pulpitis	Mild thermal discomfort	Clinical course and examination findings not fully consistent	ICOP
Pain associated with enamel crack	Visible enamel crack; referred pain; positive anaesthetic test	Most consistent with all findings	ICOP 1.1.1.1.1

Abbreviations: ICHD-3, International Classification of Headache Disorders, 3rd edition; ICOP, International Classification of Orofacial Pain.

## Data Availability

All data generated or analyzed during this study are included in this published article. Additional data are not publicly available due to patient privacy considerations but may be available from the corresponding author upon reasonable request.
